# 

**Published:** 1857-05

**Authors:** 


### NORTH AMERICAN

### MEDICO-CHIRURGICAL REVIEW.

NO. 3.—MAY, 1857.

### CONTENTS.

### ANALYTICAL AND CRITICAL REVIEWS.

PAGE

Art. I.—Des Anévrysmes et de leur Traitement. Par Paul Broca, Agrégé à la Faculté de Médecine de Paris, &c. &c., . . . . . 321

Art. II.—Historical Sketches of Quarantine. By Wilson Jewell, M.D., 356

Art. III.—Medical Notes and Reflections. By Sir Henry Holland, Bart., M.D., F.R.S., &c., . . . . . . . . . . 368

Art. IV.—Report on the Causes which impede the Progress of American Medical Literature. By S. D. Gross, M.D., &c. &c., . . . 379

### ORIGINAL COMMUNICATIONS.

Art. I.—On the Influence of Electrical Fluctuations as a Cause of Disease. By A. Littell, M.D., Surgeon to Wills Hospital, Philadelphia, . . . . . . . . . . . . . . 390

Art. II.—Stricture of the Colon. Amussat's Operation. By G. R. Henry, M.D., of Burlington, Iowa, . . . . . . . . . 409

Art. III.—On the Use of Quinoidine as a Substitute for Quinia in Miasmatic Fevers. By B. F. Graham, M.D., of Wake Co., N. Carolina, 412

Art. IV.—Extraction of a Piece of Lead-pencil from the Bladder by Perineal Incision. By S. D. Gross, M.D., Prof. of Surgery in Jefferson Med. College, Philadelphia, . . . . . . 413

Art. V.—Case of Scirrhus of the Liver in a Child three months old. By S. W. Gross, M.D., of Philadelphia, . . . . . . 414

Art. VI.—Mortality of Philadelphia for the quarter ending April 1, 1857. By Wilson Jewell, M.D., . . . . . . . . . 415

### BI-MONTHLY PERISCOPE.

#### SURGERY.

1. Inverted Toe-nail Treated without Operation, . . . . . . 424
2. Lagophthalmos from Paralysis of the Orbicularis Palpebrarum, . . 426

3. Spina Bifida. Three Cases from the same Mother, . . . . . 428
4. Removal of Tumors by a New Method, . . . . . . . . 429
5. New Mode of Employing the Taxis in Hernia, . . . . . . 429
6. Iodine Injections in the Treatment of Ovarian Dropsy, . . . 429
7. Senile Gangrene, . . . . . . . . . . . . . 431

PRACTICAL MEDICINE.

1. On the Treatment of the Night Sweats of Consumption, . . . 433
2. Treatment of Poisoning by Strychnine, . . . . . . . 434
3. Prevention of Pitting in Small-Pox, . . . . . . . . 434
4. Medicinal Effects of Ammonia and its Preparations, . . . . . 435
5. Treatment of Apnœa, . . . . . . . . . . . . 436
6. Treatment of Hooping Cough by Enemata of Assafœtida, . . . 438

PATHOLOGY.

Influence of Variations of Electric Tension as a Cause of Disease, . . 439

THERAPEUTICS AND MATERIA MEDICA.

1. Strychnine; its Uses and Abuses, . . . . . . . . . 444
2. Arsenical Poisoning, . . . . . . . . . . . . . 446
3. Absorption of Medicinal Substances from the Large Intestine, . . 447
4. Nitrate of Potash in Ascites and Anasarca, . . . . . . . 448
5. Chlorate of Potash in Membranous Formations and Ulcerations of the Mouth and Throat, . . . . . . . . . . . . . 448
6. Jelly of Iceland Moss and Cod-Liver Oil, . . . . . . . 450
7. Mode of Administering Iodine in Large Doses, . . . . . . 450
8. Tincture of Iodine for Vomiting in Pregnancy, . . . . . . 451
9. Iodine Enemata in Dysentery, . . . . . . . . . . 451

OBSTETRICS.

1. Introduction of Ice into the Uterus, . . . . . . . . 451
2. Menstruation proved to be Ovulation accompanied by Exfoliation of Lining Membrane of the Uterus, . . . . . . . . . 453
3. Diagnostic Value of Hemorrhage in Cancer of the Uterus, . . . 454
4. Sterility dependent on Diseases of the Rectum, . . . . . . 455
5. Discussion before the Paris Academy on the Treatment of Ovarian Cysts, . . . . . . . . . . . . . . . 456
6. Arrest of Mammary Secretion by the Application of Belladonna, . . 463

EDITORS' TABLE, . . . . . . . . . . . . . . 463

NECROLOGY.

Dr. Samuel Metcalfe, . . . . . . . . . . . . . 477
Dr. S. P. Hullihen, . . . . . . . . . . . . . 478
Dr. E. K. Kane, . . . . . . . . . . . . . . 478
Dr. Joseph G. Nancrede, . . . . . . . . . . . . 478

BIBLIOGRAPHICAL RECORD, . . . . . . . . . . . 480

